# 2128 Using real-time functional magnetic resonance imaging (fMRI) neurofeedback as a tool for demonstrating therapeutic efficacy in cognitive behavioral therapy

**DOI:** 10.1017/cts.2018.147

**Published:** 2018-11-21

**Authors:** Kathryn Dickerson, Katherine E. MacDuffie, Jeff MacInnes, Kari M. Eddington, Timothy J. Strauman, R. Alison Adcock

**Affiliations:** Duke University

## Abstract

OBJECTIVES/SPECIFIC AIMS: The purpose of this study was to provide individuals who have experience with cognitive behavioral therapy (CBT) with a demonstration of how using their therapeutic strategies affects their brain activity. Two challenges that face CBT and other cognitive therapies are (1) sustaining the gradual, incremental behavioral changes characteristic of the treatment and (2) measuring associated biological changes. These challenges may impede treatment efficacy and may negatively affect treatment outcomes, including patient discontinuation of CBT. Ideas for addressing these issues include providing patients with (1) a more immediate indicator of therapy effectiveness as well as (2) a biological index of behavioral change. In this study, we aimed to provide participants with an index of biological change based on therapeutic experiences via use of real-time functional magnetic resonance imaging (rtfMRI) neurofeedback. METHODS/STUDY POPULATION: We recruited participants who had already completed cognitive therapy as part of a clinical trial for depression at the University of North Carolina at Greensboro (n=13). In the present experiment, participants were asked to provide a list of negative autobiographical memories or worries as well as cognitive strategies they use to cope with negative moods. The task consisted of COUNT, MEMORY, and STRATEGY trials (30 s each). During baseline COUNT trials, participants counted backwards (e.g., 300–4). During MEMORY trials, they viewed phrases previously developed describing their negative autobiographical memories/worries. During STRATEGY trials participants viewed a strategy they use to help them process the memory/worry. First, a localizer run was completed to determine a unique region of interest for each participant. We identified peak activation within the cingulate cortex to the contrast of MEMORY (STRATEGY+COUNT). Although the task was the same, no neurofeedback was displayed during the localizer run. During the feedback runs, participants were shown neurofeedback from the cingulate cortex following both the MEMORY and STRATEGY trials. This activation was represented on a signal bar display and represented the average cingulate activation during the trial. Unlike many rtfMRI studies, the purpose here was not for participants to interact with the neurofeedback directly. Rather, a feedback summary was shown to participants after each MEMORY and STRATEGY trial as an index of how brain activity changed in response to negative memories/worries and therapeutic strategies. Our goal was not for participants to learn to self-regulate the cingulate cortex, but rather to provide participants with a metacognitive demonstration of strategy efficacy. Participants were given detailed instructions regarding the task design, the role of the cingulate cortex in depression, as well as the hypothesized direction of activation during the MEMORY and STRATEGY phases to help them interpret the neurofeedback. RESULTS/ANTICIPATED RESULTS: Results revealed that “stronger neurofeedback” (defined as the difference between STRATEGY vs. MEMORY trials) correlated with self-reported strategy efficacy ratings immediately following the scan session (*p*<0.05). More importantly, stronger neurofeedback predicted both self-reported strategy efficacy and frequency of use 1 month following the MRI session (*p*<0.05). Importantly, this relationship was specific to only those strategies used inside the scanner; and no such relationship was observed at baseline. Neuroimaging results revealed that during the MEMORY phase, activation within inferior frontal gyrus and supramarginal gyrus correlated with baseline BDI score (whole brain, cluster corrected with FSL Flame 1 to *p*<0.05). During the STRATEGY phase, the periaqueductal gray nucleus, insula, and temporal pole predicted self-reported frequency of strategy use 1 month post-scan session (whole brain, cluster corrected with FSL Flame 1 to *p*<0.05). DISCUSSION/SIGNIFICANCE OF IMPACT: We believe this study holds promise to provide a powerful demonstration for individuals that strategies used to cope with negative moods can produce significant changes in their brain.

